# Increased self-efficacy: the experience of high-intensity exercise of nursing home residents with dementia – a qualitative study

**DOI:** 10.1186/s12913-015-1041-7

**Published:** 2015-09-14

**Authors:** Cecilie Fromholt Olsen, Elisabeth Wiken Telenius, Knut Engedal, Astrid Bergland

**Affiliations:** Faculty of Health Sciences, Oslo and Akershus University College of Applied Sciences, Oslo, 0130 Norway; Oslo and Akershus University College of Applied Sciences, Oslo, 0130 Norway; Oslo university Hospital, Ageing and Health, Norwegian Centre for Research, Education and Service Development, Oslo, 0424 Norway

## Abstract

**Background:**

There has been increasing interest in the use of non-pharmacological interventions, such as physical exercise, to improve the well-being of nursing home residents with dementia. For reasons regarding disease symptoms, persons with dementia might find it difficult to participate in exercise programs. Therefore, it is important to find ways to successfully promote regular exercise for patients in residential care. Several quantitative studies have established the positive effects of exercise on biopsychosocial factors, such as self-efficacy in older people; however, little is known regarding the qualitative aspects of participating in an exercise program among older people with dementia. From the perspective of residents, we explored the experiences of participating in a high-intensity functional exercise program among nursing home residents with dementia.

**Methods:**

The participants were eight elderly people with mild-to-moderate dementia. We conducted semi-structured interviews one week after they had finished a 10-week supervised high-intensity exercise program. We analyzed the data using an inductive content analysis.

**Results:**

Five overreaching and interrelated themes emerged from the interviews: “Pushing the limits,” “Being invested in,” “Relationships facilitate exercise participation,” “Exercise revives the body, increases independence and improves self-esteem” and “Physical activity is a basic human necessity—use it or lose it!” The results were interpreted in light of Bandura’s self-efficacy theory. The exercise program seemed to improve self-efficacy through several mechanisms. By being involved, “being invested in” and having something expected of them, the participants gained a sense of empowerment in their everyday lives. The importance of social influences related to the exercise instructor and the exercise group was accentuated by the participants.

**Conclusions:**

The nursing home residents had, for the most part, positive experiences with regard to participating in the exercise program. The program seemed to increase their self-efficacy through several mechanisms. The instructor competence emerged as an important facilitating factor. The participants emphasized the importance of physical activity in the nursing home.

## Background

Dementia is a global public health problem. Each year, 7.7 million new cases of dementia are identified worldwide [1]. Prince et al. [2] estimated that 35.6 million people suffer from dementia worldwide, with likely increases to 65.7 million by 2030 and 115.4 million by 2050. Approximately 75 % of the elderly living in nursing homes suffer from dementia [3], which is also the case in Norway [4].

Physical activity is globally recognized as a positive health influence across all ages [5]. Despite this awareness, nursing home residents spend up to 94 % of their time sitting or lying down, although the residents are capable of participating in independent or assisted activities [6, 7]. A growing body of literature has suggested that physical exercise has beneficial effects on the physical [8] and cognitive [9] functions in healthy older adults, as well as in individuals with cognitive impairment [10]. Additionally, several recent clinical trials [11, 12] and systematic reviews [13–15] have demonstrated that individuals with dementia can receive the beneficial effects of physical exercise. A recent meta-analysis identified 16 randomized, controlled trials of exercise interventions in 937 individuals with dementia, supporting evidence that exercise improves the ability to perform basic activities of daily living, such as eating, dressing, bathing, using the toilet, and transferring from bed to chair [16]. For older people living in residential care facilities, regular exercise can reduce activity limitations [12, 17–19], maximize independence [12, 18, 19], likely slow the progression of dementia [12, 20], promote sleep [19, 21], and enhance the quality of life and well-being [19, 22]. A recent study showed that good muscle strength and balance were the most important physical performance variables associated with good quality of life for nursing home residents with dementia [23]. The authors stated that additional studies should be conducted to examine how to improve physical functioning and quality of life in this group [23].

In a recent literature review related to living with dementia, Murphy et al. [24] stated that until the 1990s, the perspectives of persons with dementia were largely ignored in dementia research. The perceptions of persons with dementia were difficult to assess [24], and the inclusion of their perspectives was obstructed by prior judgments that one could not rely on the testimony of persons with dementia [25, 26]. Murphy et al. [24] suggested that carers without dementia could not credibly construct the reality of living with dementia. If the experience and meaning of living with dementia are to be understood, the inclusion of persons with dementia as participants in research studies is essential. Valuing the unique insights of a person with dementia was therefore observed as a validation of persons with dementia. Providing the person with dementia an opportunity to participate in dementia research was deemed to be essential to addressing their vulnerabilities [27–29]. In another literature review, van Baalen et al. [30] emphasized that people with mild-to-moderate dementia are able to talk with clarity and insight about their experiences concerning quality of care. A successful subjective evaluation seems to depend on a minimum level of orientation to place, attention and language skills in the person with dementia. van Baalen et al. [30] concluded that measuring the quality of care from the perspective of people with dementia is in its initial phase and that additional research is warranted. Qualitative research has played a crucial role in improving our understanding of dementia and its impact on individuals, carers, families, and the broader community [31–33]. As our population ages, the impetus for improving dementia care is increasing [34]. Qualitative research is well suited to meet this call [31, 32, 35].

There has been an increasing interest in the use of non-pharmacological interventions to improve dementia symptoms and the well-being of residents with dementia and their carers [3]. One non-pharmacological intervention is physical exercise. Persons with dementia might find it difficult to participate in exercise programs because they depend on assistance during the exercise sessions. Therefore, it is important to successfully promote regular exercise in residential care facilities by encouraging the residents to participate in the available exercise programs and physical activity options. One recent study explored the experience regarding a high-intensity functional exercise program of older people who live in residential care facilities and who are dependent on others in performing daily activities [36]. To the best of our knowledge, no previous qualitative study has explored the participation of nursing home residents with dementia in a high-intensity functional exercise program. Therefore, the aim of this qualitative study was to explore the positive and negative experiences of a high-intensity functional exercise program in nursing home residents with dementia, from the perspective of the residents.

## Methods

### The participants

This qualitative study followed a pilot study for a randomized controlled clinical trial (RCT) of the High-Intensity Functional Exercise (HIFE) program [18, 37] in an urban nursing home in Norway. The nursing staff at the nursing home located eligible participants and provided information regarding the study. A convenience sample of twelve elderly people with mild-to-moderate dementia participated in the pilot study. The same twelve people also agreed to participate in the subsequent qualitative study. However, only eight informants ended up being interviewed because the study reached saturation. The inclusion criteria were as follows: having mild or moderate dementia, as defined by a score of 1 or 2 using the Clinical Dementia Rating (Table 1). The characteristics of the informants are described in Table 1.

**Table 1:** Characteristics of the participants (*n* = 8)

Participant number	1	2	3	4	5	6	7	8
Age (year)	92	88	69	92	96	86	92	87
Gender	Woman	Man	Woman	Woman	Woman	Woman	Woman	Woman
CDR^a^	1	1	1	2	2	2	2	1
QUALID^b^	17	18	29	17	30	15	15	19
Cornell	10	2	21	6	7	2	6	2
Barthel Index^c^	12	19	9	12	16	15	8	10
Use of walking aid	Rollator	Wheel chair.	Rollator	Not using	Rollator	Rollator	Not using	Not using
Berg Balance Scale^d^	46	1	16	51	24	30	45	51
Timed Up and Go (sec)	17.81	127,28	21,54	13,79	58,42	161,22 s	33,01	19,4
Attendance^e^	26	26	30	21	26	14	14	27

^a^Cognition was measured by the Clinical Dementia Rating Scale (CDR)

^b^The quality of life in late-stage dementia scale: total score range from 11 to 55. A lower score indicates a higher quality of life. Cornell Scale for Depression in Dementia (Scores totaling twelve (12) points or more indicate probable depression)

^c^Consist of 10 activities focusing on the patient’s level of dependence on help. The scores range from 0 (completely dependent) to 20 (independent)

^d^The total score ranges from 0 to 56

^e^Number of exercise sessions

### The physical exercise program

The exercise program was the HIFE program developed in Umeaa, Sweden [18, 37]. Each session included five minutes of warm-up, at least two strengthening exercises for the muscle in the lower limb and two balance exercises. The total duration of each session was 50–60 min. All of the exercises, conducted in small groups, were individually adapted, instructed and supervised by a physiotherapist. The intensity of the strengthening exercises was aimed at achieving 12 repetitions maximum (RM). The balance exercises were intended to be “highly challenging,” which meant that the balance exercises challenged the participants to reach their limits of postural stability both in static and dynamic tasks, such as throwing balls, stepping over obstacles and reaching for objects while standing. The difficulty of each balance exercise was increased, for example, by standing or walking on a more challenging surface. The physiotherapists maintained detailed records of all of the exercise sessions and documented the intensity of the exercises performed at each session. The participants exercised three times a week for 10 weeks. Table 1 shows information regarding how many times each patient participated in the exercise program. No patient exhibited any adverse effect during the exercises. The physiotherapists had been trained in the HIFE program. The importance of targeting high intensity and use of weighted belts was emphasized.

### Measurements

To describe the functional characteristics of the participants, we conducted the following assessments at baseline and 10 weeks after baseline. The instruments were chosen because this was a pilot study for an RCT and testing was used to determine the feasibility of outcome measurements.

*Balance* was measured using *the Berg Balance Scale*. The test assessed performance using a 5-level scale from 0 (cannot perform) to 4 (normal performance) in 14 different tasks involving functional balance control, as well as transferring, turning and stepping. The total scores ranged from 0 to 56 [38].

*The Timed Up and Go* was used for measuring *functional mobility*. The tester measured the time it took for a person to rise from a chair, walk three meters at a comfortable pace, turn and walk back to the chair and sit down. We allowed a practice trial and we recorded the results of the subsequent trial [39].

To measure the patients’ dependence/independence in the *activities of daily living* (ADL), we employed *the Barthel Index*. The scores ranged from 0 (completely dependent) to 20 (independent) [40].

We used *the Clinical Dementia Rating Scale* to validate the patients’ diagnosis of *dementia* [41–43].

*The Cornell Scale for Depression in Dementia* [44] was used to assess *depression* in the participants. Scores totaling 12 points or more indicated probable depression.

*“The quality of life in late-stage dementia scale*” (QUALID), a proxy-rated scale, was used to measure the *quality of life*. The informants were professional caregivers who knew the patient well and had spent at least three of the last seven days with the patient. Likely scores ranged from 11 to 55. A lower score indicated a higher quality of life [45].

The participants represented different ages (age range, 69–96 years). Three participants were able to walk without walking aids. Their scores on the different outcome measurements are presented in Table 1.

### Interviews

The semi-structured, face-to-face interviews were conducted one week after the last physical exercise session to reduce recall bias. When possible, the interview took place in the participants’ rooms at the nursing home. An interview guide developed for this study (Table 2), as well as pictures of all the exercises, were used. The pictures were shown during the interviews.

**Table 2:** Key questions forming the semi-structured interview schedule

Question 1	Have you noticed any positive effects from doing the program? (Showing the picture of the different tasks)
Question 2	Have you noticed any negative effects from doing the programme? (Showing the picture of the different tasks)
Question 3	What did you like about the program? (Showing the picture of the different tasks)
Question 4	What did you not like about the program? (Showing the picture of the different tasks)
Question 5	How easy/hard was the program? (Showing the picture of the different tasks)
Question 6	What motivated you to participate in the program? (Showing the picture of the different tasks)
Question 7	Would you like to continue a regular training program? (Showing the picture of the different tasks)
Question 8	Do you have any other feedback/anything else you would like to say?

The interviews lasted approximately one hour. We taped all of the interviews, and the secretarial staff transcribed the tape recordings verbatim. The data were analyzed using an inductive content analysis. The inductive approach enables researchers to identify key themes in the area of interest by reducing the material to a set of themes or categories [46]. The intention was to provide a compact yet general description of the phenomenon under investigation. The themes emerged from the raw data by repeated examination and comparison. Using the analysis process, we experienced an increased understanding of the material describing the participants’ experiences with the exercise program.

To ensure transparency and reliability, two researchers read the transcripts independently several times. This reading was performed with an open mind, in the interest of grasping the participants’ own views on the subject. An additional analysis was performed using the following procedures: 1) The transcripts were read to gain a contextualized impression of the text and previous preconceptions were highlighted. For this part of the analysis, the hermeneutic approach was obvious because various preconceptions played a part in our understanding. 2) Units of meaning were identified and coded. Inter-rater agreement on the codes was high between the authors. 3) The meaning in the coded groups was then condensed. 4) The descriptions reflecting the participants’ experiences were generalized into categories [47, 48]. A consensus regarding the categorization of statements and emerging themes was reached by discussion among all of the authors.

### Ethics

The study was approved by the Regional Committee for Medical Ethics in Norway. Written and verbal information regarding the study were given to the patients and their relatives by their primary caregiver. The participants provided their consent to participate in the study, often together with that of their next-of-kin, and they were informed that they could refuse to participate at any stage in the study.

## Results

**Fig. 1:**
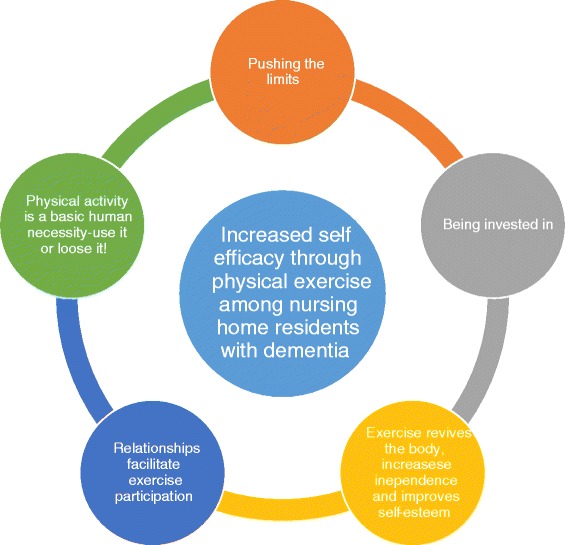
An empirical model of nursing home residents’ experiences of increased self- efficacy through participating in a high-intensity physical exercise program

The following five overreaching and interrelated themes emerged from the interviews: 1) *Pushing the limits* 2) *Being invested in* 3) *Relationships facilitate exercise participation* 4) *Exercise revives the body, increases independence and improves self-esteem* and 5) *Physical activity is a basic human necessity—use it or lose it!* (see Fig. 1). Selected quotations from the participants are presented to support and illustrate the five themes.

1. Pushing the limits

Overall, we found that all of the participants reported positive experiences with the exercises. The HIFE program contains high-intensity exercises; however, the participants did not find the exercises overly intense. They believed that the exercises were challenging but fun. They said that the feeling of exhaustion after being physically active was much better than being exhausted from lack of activity. They thought that the exercises were meaningful and relevant for everyday living. One participant emphasized the following point:

“*The exercises were challenging and seemed relevant to our needs in everyday living”.*

Another participant described her own feelings regarding the intensity of exercise in the following manner:

*“It feels good to exert yourself. Maybe I should have pushed myself even harder. We who are old may not be used to it. There is no harm in exerting oneself. Maybe it is more fun that way. It will lead to more energy and optimism”.*

A third participant added:

*“I believe that it is healthy to exercise. It is much better to be tired after exercises than be tired after doing nothing”.*

All of the participants had positive attitudes toward exercise; however, some equivocal statements appeared concerning the undertaking of the exercise sessions. They thought it was important that attendance is voluntary and that no one should be pressured into participating. Several participants felt that exercising three times a week was excessive, and they made the following statements:

*“No one should be forced to participate in exercise sessions. Either way, that did not happen, and it seemed everything went smoothly”.*

*“Twice a week is sufficient. I think three times is too much”.*

2. Being invested in

Several participants emphasized that the exercises introduced a feeling of being invested in and being noticed. Additionally, the sense of having an activity to attend to and the feeling of being useful was important. The participants explained that the exercises were important for staying healthy and maintaining everyday function.

The following statements illustrate this perspective:

*“I thought it was important because I experienced being seen and felt that I was being invested in”.*

*“As an old person in a nursing home, you experience that nothing is invested in you. We were given the impression that what we did would influence our health and everyday living. It gives me somewhat satisfaction that I can still engage in activities. It does. I mean, if I am still engaged in activities, it means that I have not given up”.*

It seemed that the capability of conducting the exercises and completing an exercise session provided the participants with a feeling of achievement and a sense of empowerment. This feeling seems to be a particularly important element. One participant explained:

*“I thought that the exercises were challenging. I was somewhat proud of myself for making it. Obviously, you should be motivated and experience capability. We got the feeling: we can do this”.*

*“… to have something to attend. When we know that something awaits us, that we are useful to them. That is very important. You cannot just walk around with the impression that no one is thinking about you. That cannot happen. We experience a sense of importance when we exercise”.*

3. Relationships facilitate exercise participation

The relationships both with other residents as well as the physical therapist seemed to facilitate exercise participation. Several participants noted the positive aspects of exercising together with other residents. They explained that watching and interacting with other participants were motivating and that the participants were positive role models for each other. Two participants elaborated on this point:

*“I experienced a positive encounter with the staff that was not characterized by detachment but rather vitality”.*

*“It is also important that we who are older demonstrate to each other that we can do this and that we are positive role models for each other. We must make this happen! It is positive for our togetherness. We were able to see that the others exerted themselves and were motivated to do the same and sometimes we were surprised what we could do ourselves and what others could do”.*

The participants emphasized the therapist’s ability to adjust and accommodate the exercises to the participants during the sessions. Older people are heterogeneous, and one cannot make the same demand on everybody. Knowledge about elderly people was noted to be an important factor. The therapist must possess this knowledge, be observant, and be able to make adjustments to the exercise program while instructing. The participants made the following comments:

“*Knowledge about the aging body is central. One should know what to do with each individual and make sure that they practice things they find difficult. The person who is instructing must pay close attention and observe how the person is doing, examine if he/she is struggling or grumbling a lot. In that case, they should not push them. That is unnecessary”.*

The communication between the therapist and participant also seemed important to the residents. A therapist with extended knowledge about elderly people created feelings of security and trust. The participants emphasized that it was nice to be met with positive expectations. The information that the therapist gave about the exercises was also appreciated. They thought it was important to learn about the purpose of the exercise. The therapist should be understanding and able to communicate with older people. The participants stated the following:

*“She seems secure, accepting and knowledgeable. It was nice to be met with positive expectations. Her positive expectations were appropriate without overwhelming us”.*

*“Yes, she knew a lot. That is important. She gained our trust. She taught us about muscles and other things that we use when we engage. It was very encouraging”.*

*“Human knowledge is important, and our instructor was good at communicating with other people”.*

4. Exercise revives the body, increases independence and improves self-esteem

The residents experienced a positive change after exercising. Several participants noted that exercising improved their mood and boosted their self-esteem. They felt that their body became more alive and vital, their energy level increased and they were more content. The following two quotes illustrate this perception:

*“I felt more like a normal person. I was happier. I had more energy to engage in conversations and I also became more positive towards other people”.*

*“I feel energized by the exercises, I am no longer sitting lethargically in a chair. I was proud of myself after the exercises. The body was more present, and that is a good feeling”.*

Several residents noted that they felt that their independence improved, and they felt less dependent on the nurses after exercising. This feeling positively influenced their self-esteem. One resident elaborated in the following manner:

*“To be able to do as much as possible independently is positive for the self-esteem, at least to me. I think it is important to exercise at the nursing home to maintain independence. Most elderly would like to be able to take care of themselves as long as possible”.*

Exercising had a positive effect on motor function and ADL performance. The participants reported that it was easier to rise from a chair, to walk and to climb the stairs after engaging in exercise. One participant had the following experiences:

*“You notice that the exercise has positive effects on both body and mind. It is easier to get up from the chair, it is easier to walk, and you can manage more in everyday life. The stair-climbing exercises got easier”.*

Exercise increased the feelings of security and self-esteem and improved self-efficacy, as one individual commented as follows:

*“It is important to have confidence that you are not going to fall when you do different things. I know that even if I risk taking a fall, I cannot stay seated. It is good to talk about your fears”.*

5. **Physical activity is a basic human necessity**—**use it or lose it!**

Several residents expressed a desire to be active. They appreciated that the possibilities for activities were more limited because of their functional limitations and nursing home routines. Despite these restrictions, they recognized the importance of being as active as possible. One participant explained the situation as follows:

*“… but I want to be as active as I can, and therefore it is important that the nursing home staff encourage activity and exercise”.*

One resident said that she was aware that her body had deteriorated and that she was not satisfied with the shape she was in. She noticed that her body moved more slowly and that her energy, strength and endurance levels were reduced. She commented as follows:

*“Bodily strength gives you an experience of living and not wasting away.... Even living in a nursing home, it is unnecessary to deteriorate to the extent we often see”.*

This resident did not appreciate the fact that she was becoming increasingly less active and more dependent on others during her stay in the nursing home. Several residents have commented that it was important to be able to move around independently and not rely on others. The ability to walk around provides the resident with the perception of having freedom and more options. One resident remarked as follows:

“Being physically active affects all of me. I gain confidence in myself. I experience increased control over my life even though I live in a nursing home”.

Another resident noted that it was especially important to be able to get outside for fresh air. She felt like a prisoner if she could not get outside. She expressed herself in the following manner:

*“My problem is that I am mad about being outside in the fresh air. When they talk about having to ask for permission to go outside… I feel like a Belsen prisoner”.*

The residents expressed a need for energy and strength to be able to move around. If you do not move, your energy levels and muscle strength decrease and this process will start a vicious cycle. The residents communicated that it was important to use the body to maintain function and strength: use it or lose it. One resident noted the following:

*“An important part of being an individual is to be able to take charge of your life and not live at the mercy of others. To do this, you need energy. I need energy to think and to move. I need strength to stand and walk. Muscle strength is important for us who are old”.*

Several residents thought that the staff should focus more on active participation and that they should recognize the functional ability of each individual. The importance of exercise in nursing homes must be recognized and the staff need to be good role models. Several residents argued the following point:

*“Too often the staff are not concerned with how much we can do for ourselves. We can do more than they think, if it is facilitated”.*

*“There ought to be more exercise in nursing homes. There is no tradition for physical activity in the nursing home. I think they may be worried that the old persons will take a fall or experience excessive demands”.*

## Discussion

This study provides insight into how older people with dementia experience participating in an exercise program. To date, few studies have reported on the experiences of people with dementia with regard to exercise; to the best of our knowledge, nursing home residents with dementia have never before been asked to relate their experiences of participating in a high-intensity exercise program. We will discuss the results within the framework of self-efficacy. It was unambiguous in the findings that self-efficacy was an important topic. Self-efficacy is the subjective assessment of one’s own capability to master certain tasks and situations, a sense of personal control or empowerment [49]. Central to the theory are the following four sources of information, which people draw on when they develop their self-efficacy beliefs: 1) enacted mastery experiences; 2) vicarious experiences; 3) verbal persuasion; and 4) physiological and affective states. Self-efficacy has frequently been used as a framework for understanding how older people experience exercise interventions [49, 50]. Thus, the discussion is presented under the following headings: “increased self-efficacy through exercise” and “increased self-efficacy through involvement”.

### Increased self-efficacy through exercise

One of the positive experiences reported by the participants was the feeling of achievement that is derived from performing the exercises. This corresponds with the enacted mastery experiences, which, according to Bandura [49], is the most powerful source of self-efficacy. Participating in the exercise program seemed to introduce experiences of mastery, which could have had a positive effect on the participants’ self-efficacy and self-esteem. The experience of exercising gave the participants a sense of doing something good for themselves, which in turn seemed to produce a change in emotional states. They reported feeling more “confident,” “proud of themselves” and “more positive”. According to Bandura [49], self-efficacy can have a profound effect on emotional states. Exercise can also lead to improved emotional states directly through the physiological process in the body, such as increased secretion of endorphins that improves mood [50]. Improved mood was reported by the majority of the participants.

An additional aspect that many of the participants proposed was the relevance of the exercise program to everyday life and the experience of improvement in physical functioning. The HIFE program is specifically designed for improving function. Previous studies have confirmed that this exercise program can improve physical function, such as balance and strength, and can likely improve well-being [18, 51–53]. Several studies have shown that older people may have low self-efficacy beliefs regarding their own capacity to exercise [49, 54, 55]. Improved physical function due to exercise and the experience of relevance to everyday function can cause a sense of mastery and increase self-efficacy [49]. The participants talked about the importance of *‘being able.’* In addition to improved physical function, such as stair walking and getting up from a chair, the participants reported several other bodily experiences related to participating in the exercise program. They reported that they felt more “vital,” had “increased energy,” felt “stronger” and had a better sense of “self-esteem”. According to Bandura [49], people rely on somatic information transmitted by physiological and emotional states when altering their self-efficacy. These states represent an important source of self-efficacy, especially in regard to physical capabilities.

Another positive aspect proposed by the participants was the group aspect of the exercise sessions. They enjoyed being with others in the same situation and used each other as role models, benefitting from seeing each other’s accomplishments. Bandura [49] emphasized vicarious experiences and modeling as sources of increased self-efficacy. People automatically compare themselves to others, and seeing others achieve something can increase their self-efficacy beliefs about their own capabilities. This perspective resonates well with the experiences of the participants who reported that they felt motivated by the achievements of the other participants in the group. The positive social aspects of group exercise interventions have been widely studied, and many people seem to prefer exercising in groups as opposed to exercising individually. The group experience may also serve as a motivator for exercise adherence [54, 56].

An important finding of this study is the perceived importance of certain instructor skills. The participants emphasized positive communication skills and knowledge concerning the aging body as positive instructor traits that created a sense of trust and safety. Verbal persuasion and the positive social support that one is capable of performing certain activities represent an important source of increased self-efficacy [49]. Furthermore, to be effectively influential, the persuader needs to exert credibility and establish trust [49]. Thus, knowledgeability and trustworthiness seem to be important instructor skills, which could have contributed to increasing self-efficacy for exercise and physical activity among the participants.

Moreover, the participants emphasized the importance of skills in individually tailoring the exercise program. Two central points emerge from the results. The exercises should be safe, meaning one should avoid pushing the participants too hard; however, at the same time, the exercises should be effective, meaning the exercises should be aimed high enough to give the participants a feeling of “being challenged” and having exercised. The exercise program HIFE is designed for continuous supervision of a person skilled in assessing and prescribing exercise for older people [18]. It can be argued that people with dementia, due to typical manifestations, such as memory loss, might have a greater need for supervised exercise than healthier elderly adults who might be able to perform exercises on their own with the appropriate dosage. Several studies have demonstrated that people with dementia are more prone to feelings of insecurity, thus establishing a safe and secure setting for the exercises might therefore be of particular importance [57, 58]. The issue of applying the proper dosage of exercise also has a parallel in the self-efficacy theory. According to Bandura [49], the difficulty of task is a factor in determining self-efficacy. If the task is deemed too difficult or if it is too easy, there will be no mastery experiences. The findings, therefore, suggested that the exercise instructor has an important role to play in moderating the appropriate dosage of exercises, thus serving the role as a facilitator of mastery experiences. This role seems to require skills in communicating with people with dementia as well as experience in prescribing exercise for older adults. Hence, the competence of the instructor seems to play a vital part in the experiences of participating in the exercise program and can be perceived as a facilitating factor. This perspective is consistent with a previous study on facilitators for promoting exercise attendance in older adults [56] and earlier research emphasizing professional competence as an important aspect of dementia care [59, 60].

### Increased self-efficacy through involvement

The participants in this study expressed an appreciation of being “invested in” and being informed about the reasoning behind the exercises. The participants stated that the exercise program represented something positive in an otherwise undemanding environment. They stated that being included, being met with expectations, and being informed seemed to create vitality, a sense of mastery and increased self-efficacy: “we can do more than they think, if it is facilitated”. When the participants spoke about participating in the exercise program, they related how they experienced life in the nursing home. The participants expressed a need to be physically active and stimulated, be actively involved in their own lives and receive attention. Central to the theory of self-efficacy is the importance of a sense of personal control over one’s own life. The inability to exert an influence over life’s events can create apprehension and apathy [49]. Research has shown that people with dementia can indeed express their preferences consistently, even in the advanced stages of dementia [26, 30, 61], and they should therefore be involved in decision-making regarding their lives and activities in the nursing home. The participants complained that there was too little physical activity within the nursing home environment. This factor was supported by previous studies showing that nursing home residents are physically inactive most of the day [6, 7]. Furthermore, the decline in functional health status is often mistakenly attributed to the natural biological aging process when, in fact, it is due to physical inactivity [49, 62]. We interpret this undemanding environment as being a barrier to physical activity and exercise. In light of the self-efficacy theory, such institutional constraints can negatively influence self-efficacy beliefs and well-being [49]. The participants perceived the exercise program as a positive counteraction to the physically inactive life in the nursing home, which could have contributed to an increased self-efficacy.

One factor that can stand in the way of involving people with dementia is their increasing cognitive impairment and frailty [61]. However, our results demonstrated that this factor must not lead to automatic assumptions that people with dementia cannot process or do not appreciate information and inclusion in decision-making. McCormack [63] found that for older people, actual decision-making was not as important as being informed and having their values and preferences heard and considered. Our findings showed that the participants were able to perceive the qualifications of the exercise instructor and feel more secure if things were explained to them. Appreciating a competent instructor and learning the purpose and benefits of the exercises can be interpreted as a wish of being included and taken seriously. Environmental and social factors that offer little support of expression of independence can also contribute to dependent behavior and loss of self-efficacy in the elderly [49]. It is important to recognize that even if persons with dementia are dependent on the nursing staff for help, this dependence does not necessarily lead to loss of self-efficacy. Although the ability to make decisions will vary according to the particular stage of the dementia disease and other personal and contextual factors, such patient involvement is suggested to increase autonomy and empowerment [64–66].

Our findings have suggested that nursing home residents generally appreciate participating in exercise programs and that this engagement has important implications for the provision of quality care and user involvement in dementia care. The findings of this study are consistent with the outcomes in many studies providing evidence of the positive effects of physical exercise on the physical, psychological and social aspects of life for older adults with dementia [9, 13, 14, 16]. The exercise program seemed to have an effect on all four sources of self-efficacy. By being involved, “invested in” and having something expected of them, the participants were able to gain a sense of power over their everyday lives and participating in group exercises represented one way of giving meaning and purpose to their lives.

### Strengths and limitations

A strength of this study is the exploration of the experiences of people with dementia from the perspective of persons with dementia. The majority of studies explored dementia experiences from the point of view of a family member or carer [24, 30]. Interviewing people with mild-to-moderate dementia required that the interviewer have some aids and possess certain skills. We regard the use of visual aids in the form of pictures of the exercise tasks as a positive contribution to the quality of the interviews. Our findings suggested that cognitive impairment is no reason to exclude people with dementia from research.

This study has several limitations. First, although thematic saturation was achieved with a sample size of eight participants, this small sample may not reflect the views of the larger nursing home community. Second, this study was conducted in an urban nursing home setting with Norwegian speaking residents and may not be generalizable to other settings or populations. Furthermore, in qualitative studies, the role of the researchers as producers of knowledge is important. We realize that our preconceptions of exercise and physical activity constitute an important part in the lives of elderly people with dementia in nursing homes and have influenced our interpretations.

## Conclusions

Our findings suggest that the HIFE program may lead to positive physical, emotional and social changes, including greater behavioral coherence, improved social interactions and increased well-being. Physical activity and exercise are rarely prioritized in nursing homes; however, the results of this study showed that the participants had, for the most part, positive experiences with participating in a regular group exercise program that has improved their self-efficacy. A competent instructor with knowledge of the aging body and ability to inform the participants was identified as a facilitator. Being involved and having something expected of them were important positive experiences reported by the participants. An unstimulating nursing home environment may be considered as a barrier.

This outcome is an important finding with clinical implications regarding the care of older people with dementia in nursing homes. Our findings are optimistic relative to the capacities of people with dementia. Healthcare professionals should be aware of the positive aspects of exercise and physical activity among nursing home residents with dementia.

## Abbreviations

ADL: Activities of daily living
HIFE program: High-intensity functional exercise program
RCT: Randomized controlled clinical trial
RM: Repetition maximum
QUALID: Quality of life in late-stage dementia scale
